# Community engagement to inform development of strategies to improve referral for hypertension: perspectives of patients, providers and local community members in western Kenya

**DOI:** 10.1186/s12913-023-09847-0

**Published:** 2023-08-11

**Authors:** Violet Naanyu, Benson Njuguna, Hillary Koros, Josephine Andesia, Jemima Kamano, Tim Mercer, Gerald Bloomfield, Sonak Pastakia, Rajesh Vedanthan, Constantine Akwanalo

**Affiliations:** 1 Department of Sociology Psychology and Anthropology, School of Arts and Social Sciences, Moi University, Nairobi, Kenya; 2 Department of Clinical Pharmacy & Practice, Moi Teaching and Referral Hospital, Nairobi, Kenya; 3 Academic Model Providing Access to Healthcare (AMPATH), Nairobi, Kenya; 4 Department of Medicine, School of Medicine, College of Health Sciences, Moi University, Nairobi, Kenya; 5 Department of Population Health & Department of Medicine, University of Texas at Austin, Austin, USA; 6 Department of Medicine, Duke University School of Medicine &, Duke Global Health Institute, Durham, USA; 7 Department of Pharmacy Practice & Center for Health Equity & Innovation, Purdue University College of Pharmacy, West Lafayette, USA; 8 Department of Population Health & Department of Medicine, New York University Grossman School of Medicine, New York, USA

**Keywords:** Hypertension, Referral networks, Barriers to care, Health information technology, Peer support, LMIC, Implementation research

## Abstract

**Background:**

Hypertension is the leading cause of death and disability. Clinical care for patients with hypertension in Kenya leverages referral networks to provide basic and specialized healthcare services. However, referrals are characterized by non-adherence and delays in completion. An integrated health information technology (HIT) and peer-based support strategy to improve adherence to referrals and blood pressure control was proposed. A formative assessment gathered perspectives on barriers to referral completion and garnered thoughts on the proposed intervention.

**Methods:**

We conducted a qualitative study in Kitale, Webuye, Kocholya, Turbo, Mosoriot and Burnt Forest areas of Western Kenya. We utilized the PRECEDE-PROCEED framework to understand the behavioral, environmental and ecological factors that would influence uptake and success of our intervention. We conducted four *mabaraza* (customary heterogenous community assemblies), eighteen key informant interviews, and twelve focus group discussions among clinicians, patients and community members. The data obtained was audio recorded alongside field note taking. Audio recordings were transcribed and translated for onward coding and thematic analysis using NVivo 12.

**Results:**

Specific supply-side and demand-side barriers influenced completion of referral for hypertension. Key demand-side barriers included lack of money for care and inadequate referral knowledge. On the supply-side, long distance to health facilities, low availability of services, unaffordable services, and poor referral management were reported. All participants felt that the proposed strategies could improve delivery of care and expressed much enthusiasm for them. Participants appreciated benefits of the peer component, saying it would motivate positive patient behavior, and provide health education, psychosocial support, and assistance in navigating care. The HIT component was seen as reducing paper work, easing communication between providers, and facilitating tracking of patient information. Participants also shared concerns that could influence implementation of the two strategies including consent, confidentiality, and reduction in patient-provider interaction.

**Conclusions:**

Appreciation of local realities and patients’ experiences is critical to development and implementation of sustainable strategies to improve effectiveness of hypertension referral networks. Incorporating concerns from patients, health care workers, and local leaders facilitates adaptation of interventions to respond to real needs. This approach is ethical and also allows research teams to harness benefits of participatory community-involved research.

**Trial registration:**

Clinicaltrials.gov, NCT03543787, Registered June 1, 2018. https://clinicaltrials.gov/ct2/show/NCT03543787

**Supplementary Information:**

The online version contains supplementary material available at 10.1186/s12913-023-09847-0.

## Background

Hypertension is the leading risk factor for premature death and disability globally [1]. Prevalence of hypertension in low- and middle-income countries (LMICs) is 32% [2], a high burden compounded by low treatment and control rates estimated at 30% and 10% respectively [3]. In Kenya, hypertension prevalence is 25%, with low treatment (27%) and control rates (52%) [4, 5], and is the fifth leading risk factor for death and disability [1].

Referral networks connect tiered healthcare systems between primary, secondary and tertiary health facilities, providing access to different levels of diagnosis and treatment, with the goal of decentralizing access to basic health services to lower levels, while optimizing access to specialized care at higher levels [6]. Hypertension care in Western Kenya follows a similar referral network, where stable patients can access basic services for treatment and cardiovascular disease (CVD) risk factor modification at lower levels, while those with disease complications can receive care at higher levels, moving along the continuum through up-referrals and down-referrals as required. However, the effectiveness of referral networks for hypertension care and other conditions in low-resource settings is limited by non-adherence to referrals and delays in referral completion [7–10]. Referral non-adherence ranges from 13–37% in high-income country settings [11], and 63–80% in low- and middle-income countries [9, 12], with contextual barriers being present at the patient (logistical barriers, lack of understanding of the reasons for referral, and associated costs), clinical provider (knowledge gaps and inefficient communication between providers), and health system level (poor documentation and inability to track and account for referred patients) [6].

The Strengthening Referral Networks for Management of Hypertension across the Health System (STRENGTHS) study is a cluster randomized controlled trial aimed at evaluating the effectiveness and cost-effectiveness of a combined peer and health information technology (HIT) strategy to improve referral adherence and blood pressure control in western Kenya [6]. Peer-based care approaches leverage unique patient-patient relationships in effecting behavior change to improve patient activation, health seeking behavior, and medication adherence [13–16]. HIT improves patient encounter documentation, patient tracking, and provider-provider communication [17].

The contextual factors that influence the implementation of this integrated strategy are unknown. Understanding these factors requires engagement with local communities, health workers, and patients. We report findings from our baseline contextual analysis, aimed at understanding factors influencing referral completion and implementation of our combined strategy. This type of community engagement has proved useful in health promotion, implementation research endeavors, and in policy making [18–20], as it unleashes positive collaborative energy needed to address specific health concerns.

## Methods

### Study setting

The study took place in western Kenya within the Academic Model Providing Access to Healthcare (AMPATH) program, which is an academic partnership between Moi Teaching and Referral Hospital (MTRH), Moi University College of Health Sciences (MUCHS), and a consortium of North American universities [21]. AMPATH has established a Chronic Disease Management (CDM) Program in collaboration with the Kenya Ministry of Health (MOH) to provide care for NCDs. We have enrolled over 40,000 patients with hypertension [22]. Features of the CDM program include task shifting with use of mHealth and clinical decision support [23, 24], linkage and retention programs [25], revolving fund pharmacies to ensure a reliable supply of medicines for hypertension and other NCDs across the health system [26], and incorporating the social determinants of health into care delivery [27].

The Kenyan health system has six levels of care. They include level 1, community services; level 2, dispensaries and clinics; level 3, health centers and maternity and nursing homes; level 4, sub-county hospitals and medium-sized private hospitals; level 5, county referral hospitals and large private hospitals; and level 6, national referral hospitals and large private teaching hospitals [28].

In Kenya, health financing is through the national social health insurance (National Health Insurance Fund), private insurance, donor funding, and out of pocket payments. When fares to the health facility are added to user fees, the incidence of catastrophic expenditure increases among many clients and they are vulnerable to poverty. This is especially noted among the poor, rural, elderly, and those suffering from chronic conditions [29, 30]. Most NCD diagnosis and treatment costs, even in the public sector, represent a substantial economic burden that can result in catastrophic expenditures [31]. There is evidence that patients with NCDs in Kenya have limited access to required medicines. Increasing access should be a focus of efforts to achieve universal health coverage [32].

### Conceptual framework and overall approach

The STRENGTHS intervention has been described in detail previously [6]. Briefly, the peer support component of the strategy involves peer navigators who are trained patients who provide logistical support to fellow patients in ‘navigating’ the health system, and provide treatment support [33] at each level of the referral network to support referral adherence, assist patients navigate health facilities, and provide psychosocial support. To qualify for this position, a peer has to be a patient who has well-controlled hypertension and has experience navigating the health system. A peer navigator meets with patients to review referral rationale and logistics. S/he then personally receives referred patients and walks them through the health facility ensuring they find all required services. The peer support intervention therefore enhances communication between healthcare providers and patients, helps patients navigate the health system easily, and promotes holistic wellbeing through psychosocial support [6].

The HIT component is designed to augment a pre-existing medical records system to support a referral system through facilitating data sharing by all providers and peer navigators across all levels of care, providing clinical decision support, tracking referral lists, and dashboards for monitoring referral process metrics. The HIT intervention was to support the referral system in four ways. First, it was to facilitate data sharing by healthcare providers and peer navigators across the health system. Secondly, it was to provide clinical decision support to facilitate referrals. Thirdly, the HIT would help in tracking and sharing of real-time patient referral data. Lastly, the HIT was resulting in a platform for monitoring key referral process data including referral wait times and completion rates [6].

**Fig. 1:**
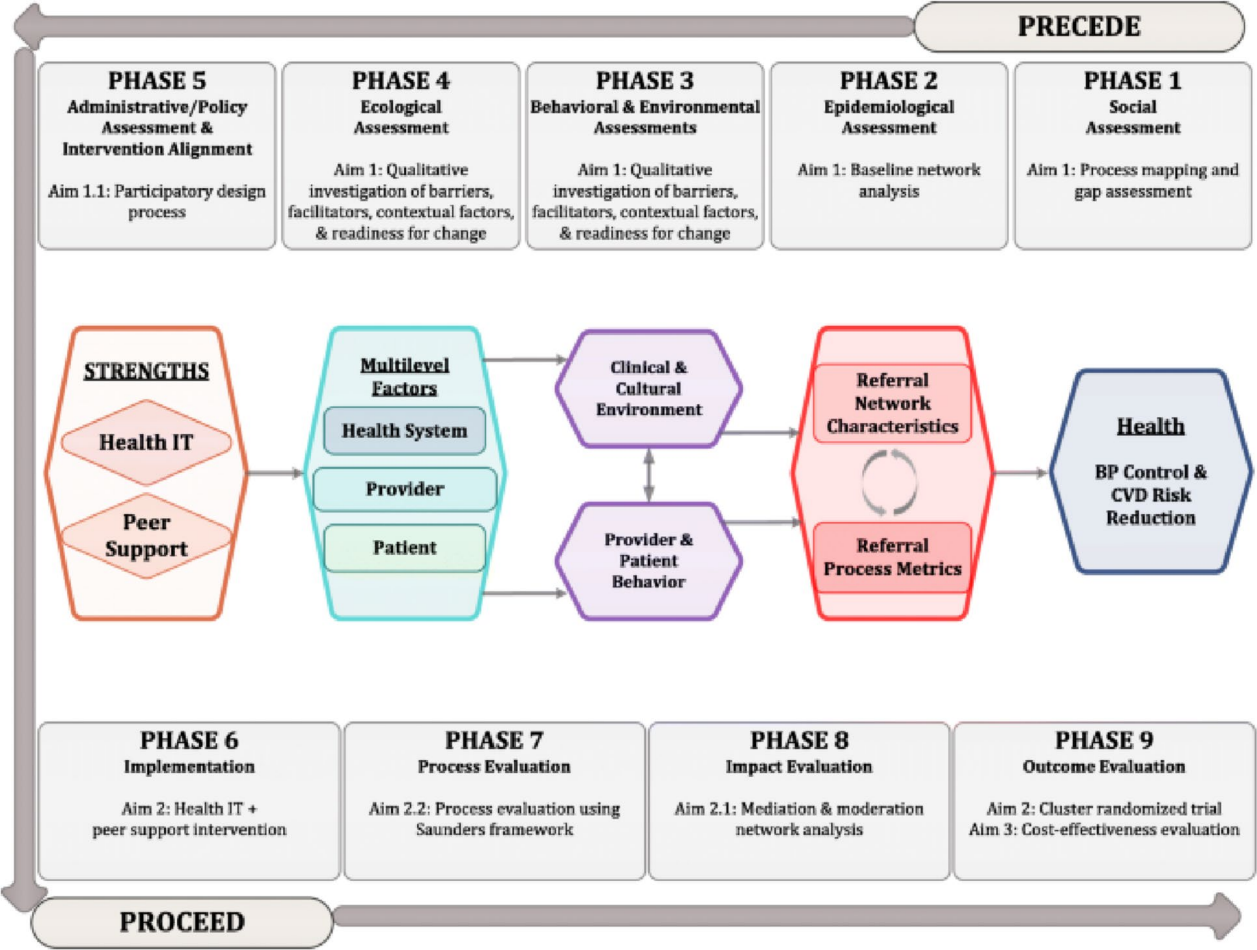
Implementation framework and conceptual model of change (Mercer et al., 2019)

We used the PRECEDE-PROCEED implementation science framework and conceptual model of change to inform our baseline evaluation, intervention refinement, study conduct and outcome evaluation (Fig. 1) [6].

The PRECEDE-PROCEED framework applies a participatory multi-pronged approach that facilitates a thorough assessment of multi-level factors that affect uptake and success of interventions. The PRECEDE part refers to the comprehensive needs assessment carried out to understand the local context and factors underlying the matter of interest. The PROCEED aspects involve use of information gathered in the earlier PRECEDE period in designing, implementing, and evaluating new interventions. There are nine key elements (Fig. 1) of this framework namely social assessment, epidemiological assessment, behavioral and environmental assessment, educational and ecological diagnosis, administrative and policy assessment, implementation, process evaluation, impact evaluation, and outcome evaluation [34].

The focus of this current manuscript was Phases 3 (Behavioral and environmental assessment) and 4 (Educational and ecological diagnosis) of the framework where we sought to understand the behavioral, environmental and ecological factors that would influence the uptake and success of the STRENGTHS intervention. The findings from this work would then be used to inform Phase 5 of the study, that is, the participatory design process.

The study was conducted within six geographically separate referral networks, each centred on a secondary-level health facility (Kitale, Webuye, Kocholya, Turbo, Mosoriot and Burnt Forest) staffed by medical officers (general practitioners who have completed medical school) and clinical officers (mid-level providers who have completed a diploma level clinical training program). Each of these six facilities serves as the link between the tertiary-level centre, staffed by specialists and several primary-level health facilities staffed by clinical officers and nurses.

We used a combination of qualitative research methods, including traditional community assemblies (*mabaraza, singular: baraza*), focus group discussions (FGDs), and key informant interviews (KII). The mabaraza allowed exploration of both community and individual perspectives, while FGDs gathered people from similar backgrounds or experiences to discuss the topics of interest [35]. KIIs were in-depth interviews with people who had first-hand knowledge on study issues and knew what was going on in their communities. All sessions were conducted between October and December 2019.

### Study materials

Interview guides were developed to ensure each data collection session raised relevant data that could inform the strategies being designed. The interview guides covered the following topics: general information on hypertension and available care in the communities, referral networks for hypertension, factors influencing completion of hypertension referrals, opinions on the proposed strategies, and readiness to take up the strategies. The guides were available in English and Swahili.

The baraza and FGD guides were tested for face validity on three community members while the KII guide was tested on three clinical staff of different cadres (clinician, data/records personnel, and administrator). During these tests, we noted that we needed to simplify the language used to ensure understanding by the participants, inform participants in advance to have them set aside the time to participate in the sessions, and organize appropriate venues within the facility or the community to conduct the sessions. These observations informed revision of the tools and overall planning, to facilitate smooth flow and active participation during the data collection period.

### Participants and recruitment

The study team worked with the AMPATH leadership, Ministry of Health (MOH) representatives, and local community leaders to organize the baraza sessions at a venue that was convenient to study participants. The two categories of FGDs (individuals with hypertension and clinical staff) were formed by purposive sampling and had 6–10 participants each. Forty-two clinical staff were recruited for the FGDs and they were from each of the three levels of the health system. They were identified as staff participating in the referral process, or those who self-identified as doing so, or those nominated by their peers as having knowledge of hypertension management and/or the referral process. Thirty-eight patients were recruited from each of the three levels of the health system by random sampling as they came to the hypertension clinic, with an attempt to balance gender. For key informant interviews, 18 clinical staff in leadership roles and health facility administrators were purposively recruited from the six clusters. All participants were approached via face-face interactions. To ensure data saturation, we collected information representative of the range of experiences and perspectives relevant to the research question, we had a 42 clinicians, 18 facility leaders, and 38 patients, and they provided meaningful information on the topic of interest.

### Data collection procedures

The conduct of mabaraza was quite similar to focus group discussions but with several key differences. Firstly, mabaraza included a large and heterogeneous group from the community. Secondly, each baraza took a longer time than an FGD. Finally, to promote community ownership, session facilitators were encouraged to take a backseat and allow the group discussion to be community member-driven as much as feasible [35].

Research assistants were competitively recruited to facilitate consenting and data collection sessions. They were recruited on basis of education and knowledge of qualitative research. They held a minimum of a diploma in health or social sciences. They were trained to ensure they had skills needed for consenting and data collection procedures. They were all familiar with the requirement for confidentiality and the protection of participants’ privacy. All data collection sessions were conducted at a venue that was convenient to the participants. The mabaraza were conducted in open fields in the Chief’s camp or in local churches. FGD for both patients and clinicians were conducted in the health facilities. On average, the mabaraza lasted 1–2 h, while the FGDs took 1 h. For KIIs, the interview locations included their offices, conference rooms, or facility observation rooms. Each of the KIIs lasted approximately 30 min.

For all sessions, a trained moderator used the semi-structured discussion guide to initiate the discussions. The dialogue was allowed to evolve as additional relevant issues emerged. For the FGDs, a scribe took notes as the session facilitators moderated the dialogue. At the end of each day, the research team conducted a debriefing session to summarize findings, compare impressions, identify procedural problems, and develop plans for future data collection sessions. All sessions were audio-recorded, and note-taking was also performed, in order to help capture information.

Verbal consent was obtained for the mabaraza and FGD participants. In Kenya, it is common for group meetings (for research or otherwise) to start with a prayer and an explanation on the meeting’s purpose. Participants are then verbally asked if they consent to participation and continuation of the gathering. Noteworthy, the baraza setting is usually much larger than a FGD because it can have 50 or more participants [35]. It is therefore common to use verbal consent during mabaraza. Written informed consent was obtained for the KIIs. All participants were 18 years or older, provided informed consent, and resided in the identified sites. All participants were provided a transport allowance and either refreshments or meal allowance.

### Data management and analysis

All mabaraza, FGDs, and KII audio-recordings were transcribed and translated into English by the research team. Deductive content analysis of the transcripts and notes was performed using NVIVO software. Each transcript was read, line by line, and specific segments were assigned codes based on content’s relevance to specific questions in the study tool. Two analysts compared codes, arrived at consensus, and developed a coding frame to guide analysis of all the transcripts. They searched for codes touching on (a) perception of factors influencing referrals for hypertension; (b) opinions on the HIT and peer navigator strategy; and (c) factors and concerns that could impact the success of the strategy. Coded items were grouped together into distinct themes and analyzed in line with study questions.

## Results

A total of 284 participants were engaged in this study across mabaraza, FGDs and KIIs, with their demographic details summarized in Table 1. There were more female participants in the mabaraza and patient FGDs while there were more male participants in the KIIs and clinician FGDs. Mean age was highest in the patient FGDs and lowest in the clinician FGDs at 60.9 years and 33.9 years respectively. The majority of participants in the mabaraza and patient FGDs had attained a maximum of a secondary education while all participants in the clinician FGDs and KIIs had attained a university or college level of education.

**Table 1:** Participants’ characteristics

**Category**	**Age (years)**	**Sex**		**Level of education**					**Total**
		**Male**	**Female**	**None**	**Primary**	**Secondary**	**College**	**University**	
	**Mean** ± **SD**	**N (%)**	**N (%)**						**N (%)**
**Mabaraza**	45.3 ± 13	91 (49)	95 (51)	2	61	96	22	5	186 (67)
**Patient FGD**	60.9 ± 10	18 (47)	20 (53)	4	23	8	3		38 (14)
**Clinicians FGD**	33.9 ± 7	27 (64)	15 (35.7)				37	5	36 (13)
**KIIs**	36.7 ± 7	12 (66.7)	6 (33.3)				9	9	18 (6)
**Total**				**6**	**84**	**104**	**71**	**19**	**284**

### Barriers perceived to influence completion of referral for hypertension

Several supply and demand side barriers were reported as influencing completion of referral for hypertension at all study sites. In the PRECEDE-PROCEED framework, predisposing factors include knowledge, beliefs and attitudes, perceptions of susceptibility and severity, self-efficacy, personal values, social norms, motivation and intention. The enabling factors that can influence health behaviors include access to resources, social support, physical environment, policies and regulations, skills and competencies, time and convenience, and socio-cultural factors [34]. In this study, the factors that were perceived as barriers to completion of referrals were reported as disabling factors. Table 2 shows themes reported by clinicians, patients and general community members.

**Table 2:** Barriers perceived to influence completion of referral for hypertension in western Kenya

	**Patients**	**Baraza**	**Clinicians**
***Demand side factors***			
**Cost related factors**			
Lack of money for treatment cost	x	x	x
Lack of money and means for transport or fare	x	x	x
Lack of money to buy medicines	x	x	x
**Referral knowledge**			
Ignorance on importance of referrals			x
Misconception about referral and referral facilities			x
Challenges in navigating the referral facility		x	
Fear that referral signaled a poor prognosis	x		
***Supply side factors***			
**Facility accessibility**			
Long distance to the referral facility	x	x	x
Poor road infrastructure	x	x	x
Bad weather conditions	x	x	x
Lack of ambulance for transport			x
**Service Cost & Availability**			
Long waiting hours before being attended to	x	x	x
Lack of specialized clinics for hypertension	x		
Expensive cost of treatment and investigations	x	x	x
Lack of specialized health providers		x	x
Shortage of health providers	x	x	
Lack of medicines for hypertension in the health facility	x	x	x
**Referral Management**			
Lack of proper protocol and tools for referrals			x
Lack of proper explanation on the need for the referral			x
Poor communication with the referral facility			x
Unwillingness of referral facilities to accept referrals			x
Lack of a written letter for referral			x

x indicates a sub-theme was discussed by patients, mabaraza, or by the clinicians

### Demand side barriers influencing referrals

As shown in Table 2, demand-side barriers included two main themes that fall within PRECEDE-PROCEED predisposing anddisabling factors: Lack of money for care, and inadequate knowledge on referrals and referral facilities. Of note, concerns about lack of knowledge on the importance of referrals, and misconceptions about it were only identified by the clinicians.

Financial constraints including lack of money for treatment cost, transport to the facility, and lack of money to buy medicines was reported as a major challenge as illustrated in a community baraza (Table 3). The cost of investigations and treatment for hypertension was noted to be high and hence not affordable to some of the patients. Sometimes patients reached the referral facilities but after paying for the diagnostic tests, they could not afford required drugs. This was frustrating even to the healthcare workers. Clinical staff from Burnt Forest explained this barrier and how burdensome it was to patients.

**Table 3:** Perceived barriers to completion of referral

Demand Side Barriers	Illustrative excerpt
Cost related factors^a, b^	*“So it is normally hard. They don’t have money. When you enter in Referral [a level 6 health facility], they tell you to pay first. At times transfer is normally hard due to finances…When you reach there you are stranded financially.” (Baraza, Mosoriot)*
	*“I think also in line with that maybe sometimes you might have done a few tests here and the patient has been referred to MTRH where he repeats the tests again… but bearing in mind that economic status of this patient, you are also burdening him. So, in the end you have done the right investigations, you have found the right diagnosis but now you cannot treat the patient because in the end the patient cannot afford the drugs. It is like zero work.” (FGD, Clinical staff, Burnt forest)*
Referral knowledge ^b^	*“People tend to believe that when you are referred to MTRH your disease is so critical that you are going to die. So, the patient will tell you he will not go to referral because that is where he will die. So some decline referral because of such beliefs. So instead of them knowing that they are going to be assisted, they believe that going there is a death sentence.” (FGD, Clinical staff, Burnt forest)*
**Supply Side Barriers**	
Facility accessibility^a, b^	*“Another thing is the weather condition (All laugh). There are those that come from areas where when it has rained, you cannot move to the next facility.” (FGD, Clinical Staff, Kocholya)*
Service cost & availability^a, b^	*“My opinion is that if it were possible, in these big hospitals…there should be a place set aside where they come to be attended… It should be set aside for you to come for pressure to be treated.” (FGD, Patient, Burnt Forest)*
	*“You can queue in the hospital then you get tired and you decide to head back home.” (FGD, Patient, Webuye)*
Referral management^b^	*“Our peripherals don’t communicate. We only know that there is a patient who had been referred, we don’t have any knowledge of a patient who is coming so that we do prior preparation to receive that patient.” (KII, Records, Kitale)*
	*“It is also about the information given to the patient in the facility. ‘Why are you being referred to that facility?” That means the patient should be given the reasons and importance of being referred, and what should be done in that hospital.” (FGD, Clinicians, Kitale)*
	*“There is also another point, like you are referring somebody and then you don’t have a point person to call on the other side… we don’t have that kind of connection, so it is a challenge.” (FGD, Clinical Staff, Kocholya)*
	*“We have some very sick patients who need to be transferred to MTRH or maybe any other place through ambulance. Usually there is lack of ambulance to ferry the patient to the other facility. This patient cannot walk, this patient is not willing to take a matatu [public van], they won’t reach there” (KII, CO, Kitale)*

^a^Reported by patients and community members

^b^Reported by clinicians

Limited knowledge on the reason for, and importance of, referral was found as another influence on the completion of referral. For instance, asymptomatic patients needed more education on why they were being referred. They needed more information on the value attached to completing the referral immediately. Furthermore, some patients had fears that being referred to a higher-level facility meant that they were likely to die. They would describe earlier cases of people who went to the same facility after referral and died. For instance, clinical staff from Burnt Forest shared about fears noted in patients referred to the national referral hospital.

### Supply side barriers influencing referrals for hypertension

Several supply side barriers to referral completion for hypertension care included four main themes that are under PRECEDE-PROCEED disabling factors (Table 1): long distance to health facilities and poor road infrastructure, low availability of services and staff, unaffordable services, and poor referral management.

Long distance and accessibility of the referral facility was a concern. Furthermore, rugged terrain, coupled with poor road networks and bad weather conditions, were reported as factors that influenced availability of transportation and consequently completion of referrals. In fact, in some areas there were times when the roads were impassable, especially during the rainy seasons (Table 3).

Other supply side barriers that could influence care and referrals were inadequate numbers of well-trained providers, lack of drugs, costly services, and inadequate numbers of clinics specialized in hypertension care. Low numbers of providers meant patients spent a lot of time at the health facilities. Patients disliked queuing for long hours. Some would give up, while others would not bother completing the referral at all.

Other barriers were broadly associated with referral management by clinicians and health facilities. There was poor documentation for referrals and the facilities lacked proper protocol and tools for referrals. This resulted in a situation where a referred patient could not be easily identified and received. Poor or lack of communication with the referral facility influenced referral completion. Moreover, provider-patient communication by referring provider was also deficient. Lack of a written letter for referral was discouraging to clients. Referred patients also wished to get information on the costs for expected services at the referral facility. They also needed full explanation on their condition and on the importance of the referral being made. They also lacked staff to accompany the patient for referral and some referral facilities could be unwilling to accept some referrals. Lack of a point person at the referral facility was also described.

Delays in making referrals as well as lack of transport services including the ambulance to the referral facility influenced completion of the referral process. Respondents reported that the ambulance service was rare to get since they were few in number. Consequently, patients lacking other means of transport would not complete the referral as expected.

### Opinions on the HIT and peer support strategies

All participants felt the integrated strategy would be well received and could improve delivery of care. Clinicians also expressed readiness of the health facilities to embrace both components of the strategy.

Patients expressed interest in participating in peer support programs and readily discussed benefits of this option (Table 4).

**Table 4:** Perceived benefits and concerns: Peer based and health information technology intervention in the STRENGTHS study

Peer based support		Illustrative excerpt
***Benefits***^a,b^	Health education though peers	*“With peer support, it is easier to educate people on the danger signs… when they are taught on the dangers, they tend to listen…” (KII, Clinician, Kocholya)*
	Motivation for positive patient behavior	*“We are being told most of the time to reduce salt, but we don’t do it. If you get a peer telling you, ‘I reduced salt and this is what happened,’ if you explain that to someone who is adding salt secretly, they will stop, because they will see your pressure is stable because you did it” (FGD, Patient, Mosoriot)*
	Follow up and reduced defaulter rates	*“It will help to reduce the number of defaulters in the village.” (Baraza, Kocholya)*
	Psychosocial support	*“I think it will be of help in terms of adherence and also just the psychosocial support because they will interact with more people who have hypertension and they will feel they are not alone” (FGD, Clinical Staff, Kitale)*
	Peers understand their lived experiences	*“You use the person who really knows where the shoes pinches, so for example, if you use somebody who is hypertensive to address hypertensive clients, they will listen, ‘There is somebody who can understand us.’” (KII, Records, Webuye)*
	Assistance in navigating the health facility	*“If maybe you are referred…he will explain to you… You won’t go there like someone blind because he will have already told you which office you will go to. [Upon arrival], you will find another one who will direct you at the referral and so there will be communication from one dispensary to the referral hospital.” (FGD, Patient, Turbo)*
	Patient advocate	*“Other people come and pass you because they are known by the medical workers. So, he will be like your advocate.” (FGD, Patient, Kitale)*
	Reminder on clinic appointments	*I think there is benefit, let’s say a woman forgets the clinic date. If there’s someone who will remind them—like two days to the clinic date, it will be very important (FGD, Patient, Mosoriot)*
***Concerns***^a,b^	Confidentiality	*“My worry is that I will be sharing health information about the condition of my body with someone that maybe comes from my location. Am concerned that he might be tempted to go and discuss with other community members how my body is weak or so” (FGD, Patient, Turbo)*
**Health information technology**		
*** Benefits***^b^	Easier communication between providers	*“They also feel good because things like [laboratory] results, you can just get in the computer, rather than sending them again to the laboratory.” (FGD, Clinicians, Kocholya)*
	Well-organized tracking of patient information	*“Mostly now days we use the tablets and most of the information is easily traced…When am referring a patient, the other person can receive a message informing them to expect a client referred on that particular day. Also during follow up, you can get information that, this client that you saw, these are the recommendations, or this is what we have done to this patient, yeah, without even seeing that particular patient. Or if there are [laboratory] results, you get them electronically within a short period…” (FGD, Clinicians, Kocholya)*
	Reduced paper work	*“You will carry a lot of information—that needed the whole of this building—within one small gadget and that information can be transferred to another point easily than getting a whole truck to transfer files of those clients.” (FGD, Clinician, Webuye)*
*** Concerns***	Sensitivity of HIT content and confidentiality^a,b^	*“This is the secret—they don’t want the type of disease revealed…You know everybody has concerns about his disease getting known—he wants it to be a secret.” (Baraza, Burnt Forest)*
	Patient consent^a^	*“One concern, a patient will say like, ‘You are making my disease be known to other people.’ Now maybe the person didn’t want other people to know about their conditions. So maybe that could be a challenge which may arise.” (KII, CO, Burnt)*
	Reduced patient-clinician interaction^a,b^	*“You come there and the clinician on the tablet all the time. You know, there is something called doctor-patient relationship…You are supposed to earn your patient’s confidence, if you are just on a machine and that reduces your interaction with the patient, that doesn’t seem to work very well…” (KII, Clinician, Webuye)*

^a^Reported by patients and community members

^b^Reported by clinicians

The peer-based components would provide an opportunity to acquire more knowledge on hypertension through health education since patients who would understand the disease better could teach their peers. They preferred having a peer – who was perceived as an insider – provide health education to them, compared to any other individual. They would get motivated through sharing of experiences, and get advice on how to better manage hypertension. The peer system would help in follow up and monitoring of individuals who have not completed their referrals, and thereby facilitate improved referral completion of patients. The peer support would provide a good opportunity for psychosocial support and encourage adherence to treatment. This would then translate into better health outcomes.

The peer support was expected to ease the discomfort patients experienced at the health facilities they were referred to. Having a peer at the health facility meant that a patient would find someone who was well informed on the disease, as well as on the facility. This would consequently result in better care visit experiences. Where providers would likely be too busy to counsel patients fully, the peer would have adequate knowledge and time to respond to any concerns the patients would have. Having someone always available to receive referred patients was seen as an important advantage of the peer component of the strategy. S/he would help them navigate the health facility, showing them where different services were available. S/he would advocate for quality care and ensure the patients are treated well and seen in good time. In addition, the peers would help patients remember their appointment dates.

The concern reported about the peer-based strategies touched on privacy and confidentiality. They argued that for patients who may not have disclosed their condition to others, they were likely to get concerned if they learned that the clinicians – who were the expected keepers of their confidential status—had shared their details with anyone else.

Regarding the HIT strategy, benefits of using it were reported by clinicians only. They noted that it would ease communication between providers, facilitate tracking of patient information, and reduce paperwork (Table 2). However, participants expressed concerns regarding the sensitivity of HIT content, patient consent, and potential for reduced eye contact with providers as they enter data into the electronic gadgets.

Participants were asked to describe any factors that would influence the implementation of the strategy. On the peer navigator side, the type of information accessible to them and their level of knowledge especially in handling the gadgets would be critical. They would need thorough training on the gadget, how to use it for the HIT strategy, and how to take good care of it in order to avoid loss of data. Availability of qualified providers and their positive attitude towards the strategy would make the HIT work.

Clinicians also noted that they were used to scribbling during the patient-provider encounter. Those with slow typing speed were therefore likely to dislike capturing of the client data on an electronic gadget. Unavailability of HIT equipment, poor internet connectivity and power shortage were discussed as potential barriers to smooth flow of the HIT strategy. Lastly, while noting that the HIT would only address hypertension referral matters, inability to use it for additional care purposes at the facilities reduced its value towards the general strengthening of integrated care provision at the health facilities. This was reported as a potential weakness of the HIT component because instead of integrating it within the system and infrastructure, it was being set up only for hypertension care.

## Discussion

This qualitative study was carried out in western Kenya to gather community input on a proposed integrated HIT and peer-based support strategy to improve hypertension referral adherence and blood pressure control in the region. Overall, participants were accepting and interested in the strategy, and saw opportunities to address key challenges to referral adherence.

We chose to use the demand versus supply side broad classification as prior literature highlighted barriers to access and utilization of healthcare services can either be viewed and addressed from the patient side (user) or the health system side (supplier) [36, 37]. Demand side barriers fell within the PRECEDE-PROCEED framework’s predisposing and disabling factors. They included the direct and opportunity costs incurred by patients in completing referrals, concerns of service unavailability either because of lack of adequate healthcare personnel, or available personnel not being able to provide the desired quality of service, and finally, a lack of awareness about how the referral system works. On the supply side, PRECEDE-PROCEED’s disabling factors were recorded including long distance and/or inadequate transport infrastructure to the referral facility, as well as ineffective communication between the referring and receiving facility have been identified as key barriers to effective referral systems.

Our study corroborated prior literature while identifying new themes. First, cost-related factors are dominant barriers to referral completion and were reported uniformly by all participant groups. This highlights a need for interventions to increase access and affordability to referral care as both direct and indirect costs were similarly reported. We also found that knowledge and comprehension of the actual referral—why patients were referred—was a barrier. Of note however, this was only reported by clinicians. This lack of understanding may create opportunities for misconceptions about the need for referral, such as the association of a referral with an impending poor prognosis, as well as lead to seeking alternative sources of care e.g. traditional medicine men. Interventions to address referral non-compliance in hypertension care will therefore need to strengthen provider-patient communication to improve clarity and comprehension on the need for referral. Finally, perceptions about service availability at the receiving facility, and the quality of available services was also identified as a barrier to referral compliance in our study – unearthing a potential intervention point to bridge the information gap for referred patients that may improve referral completion.

### Perceived benefits and concerns regarding the peer based and HIT components

Participants noted that peer support could be leveraged to assist patients overcome some of the identified barriers. A peer could help referred patients plan for timely referral completion by scheduling their visit for them, inquiring about clinic and service availability, and following up with participants as they prepared to complete their visit. In addition, participants agreed that the peer-patient interaction could be used to discuss some of the anticipated logistical challenges the patients may have and brainstorm mitigating strategies. The peer-patient interaction could also be leveraged to improve patient understanding of their disease state and need for referral. Finally, once patients arrived at the receiving facility, peers could provide navigation services so patients knew where to go to access the required service [33, 38]. Of note, a prior study identified that the lack of preferential treatment for referred patients at the receiving facility was a barrier to referral completion, a barrier that would be addressed by peer navigation services [39].

Prior literature on barriers to peer based care approaches includes role conflict – whereby the role of the peer within the ecosystem of healthcare providers is unclear, and hostilities may arise including an unsupportive work environment [40]. Our study found that while the HIT and peer-based model were acceptable and well regarded by patients, providers and general community members, there were two main concerns raised regarding the proposed strategies. First, there were apprehensions about confidentiality of patient data with the use of both the HIT and peer-based model. In this study, the risk of loss of patient confidentiality was noted if the provider were to share information about a patient’s condition to a peer, or if a peer divulged the information with other parties. Training, therefore, would be required to ensure peers understand that they have to maintain patient confidentiality and not divulge information with unauthorized persons. Secondly, there were uncertainties on the effect the use of HIT would have on clinician-patient encounters. Prior literature has highlighted the need for ensuring HIT either augment or at least do not impede patient-provider communication [41]. Similarly, participants thought that clinicians would interact less with the patient due to a need to enter data on the HIT tools.

### Incorporation of findings to intervention refinement

Our findings on factors that could affect uptake and success of our intervention were presented to a multi-stakeholder group comprised of patients with hypertension, clinicians, STRENGTHS researchers, peer health workers, health system administrators and health informatics professionals. The team utilized the findings to make adaptations to the STRENGTHS intervention through a human-centered design approach aimed at improving acceptability, appropriateness and feasibility of the final form of the intervention. We have described these adaptations in detail separately [42].

Our proposed HIT and peer based support strategy did not address all the demand and supply side problems reported in this study. Future implementation science should therefore consider the persisting barriers to completion of referral for hypertension [6]. On the demand side, strategies to alleviate financial constraints are critically important to improve completion of referrals [43]. On the supply side, the long distance to services, limited number of expert providers, stock out of supplies and long queues require attention. In addition, lack of adequate numbers of ambulances combined with the general poor transport infrastructure disables referrals for very sick patients.

### Importance of community engaged research

Understanding perceptions of patients, providers and other local populations regarding contextual factors that influence referral completion for hypertension provides an opportunity for development of locally acceptable and appropriate strategies. Community engaged research implies a process whereby researchers work collaboratively with communities of research during all or several stages of the research program; the ‘researched’ are allowed space for extensive participation and decision making, for instance, they are involved during identification of the research problem/s, implementation of the study, and dissemination of findings [44, 45]. Community engagement activities in research include having an advisory board/group of community representatives; obtaining input from community representatives during the study; collecting data in locations beyond main university centers; involving community representatives when recruiting and retaining study participants; sharing study findings with local communities; and engaging community representatives when translating findings into practice or policy [45, 46]. It is important to engage users of any proposed strategy early – during the pre-design formative phase, and thereafter during every step of strategy design and implementation [47–49].

Research has shown hypertension is burdensome globally and novel ways of managing patients are urgently needed, including health information technology and peer-based support strategies. By listening to concerns from patients, providers and general community members, the STRENGTHS study incorporated the voices of locals into the emerging strategy. The benefits of this approach are numerous as evidenced by community-engaged research, an approach to research designed to improve health through involvement of individuals from the community of research in shaping the research activities. The research team fully appreciated experiences and indigenous knowledge of community members, and treated them as co-creators of knowledge in the research [49–51]. This approach allows for exchange of deeply informed understanding of culturally and context specific information that consequently facilitates design and implementation of acceptable, useful, and scalable health programs [35, 52].

## Study limitations

A strength and also limitation of our study is its qualitative design; therefore, our findings may not be generalizable to other settings as the contexts may be different, and subsequently, perceptions about referral care, or the peer and health IT intervention by different stakeholders may be different. In addition, we did not selectively engage patients who had prior experience with referral care, such that some of them may not have actually ever been referred. Findings may be different if only patients who had prior experiences with referral care were recruited.

## Conclusion

Patients with chronic conditions such as hypertension require consistent management and efficient referral up and down the tiers of health care. This study analyzed opinions of patients, providers and general community members on barriers influencing completion of referral for hypertension. They also reflected on the value of the STRENGTHS HIT and peer-based support system to improve these referrals. All participants felt the strategies could improve delivery of care and expressed readiness for it; however, they also shared concerns to be considered before implementation. Our findings contribute to literature on importance of community-engaged research when developing and implementing strategies to improve referral of patients in low- and middle-income countries. Appreciation of local opinions and patients’ realities is critical to development and implementation of hypertension referral strategies. We anticipate that the lessons learned and reported here will be useful for similar chronic disease referral programs worldwide.

### Supplementary Information

**Additional file 1.**

## Data Availability

All study tools, transcripts and coding frames used in this study are available from the corresponding author on reasonable request. The data are available provided a robust data management plan is provided to the corresponding author and the request for data is approved by the author's institution. The data are not publicly available due to concerns about confidentiality.

## Abbreviations

AMPATH: Academic Model Providing Access to Healthcare
CVD: Cardiovascular Diseases
FGDs: Focus Group Discussions
HIT: Health information technology
Hy-TREC: Hypertension Outcomes for T4 Research within Lower Middle-Income Countries
IREC: Institutional Research and Ethics Committee
KII: Key Informant Interviews
LMICs: Low and middle-income countries
MOH: Ministry of health
MTRH: Moi Teaching and Referral Hospital
MUCHS: Moi University College of Health Sciences
NACOSTI: National Commission of Science and Technology
NIH: National Institutes of Health
NHLBI: National Heart, Lung, and Blood Institute
STRENGTHS: Strengthening Referral Networks for Management of Hypertension across the Health System
